# Prognostic significance of KIF2A and KIF20A expression in human cancer: A systematic review and meta-analysis

**DOI:** 10.1097/MD.0000000000018040

**Published:** 2019-11-15

**Authors:** Xing Li, Kunpeng Shu, Zhifeng Wang, Degang Ding

**Affiliations:** a Department of Urology, People's Hospital of Zhengzhou University; b Department of Urology, Henan Provincial People's Hospital, People's Hospital of Zhengzhou University, School of Clinical Medicine, Henan University, Zhengzhou, Henan, China.

**Keywords:** cancer, KIF20A, KIF2A, meta-analysis, prognosis

## Abstract

**Background:**

The kinesin family (KIF) is reported to be aberrantly expressed and significantly correlated with survival outcomes in patients with various cancers. This meta-analysis was carried out to quantitatively evaluate the prognostic values of partial KIF members in cancer patients.

**Methods:**

Two well-known KIF members, KIF2A and KIF20A, were investigated to evaluate their potential values as novel prognostic biomarkers in human cancer. A comprehensive literature search was carried out of the PubMed, EMBASE, Cochrane Library, and Web of Science databases up to April 2019. Pooled hazard ratios (HRs) and odds ratios (ORs) with 95% confidence intervals (CIs) were calculated to assess the association of KIF2A and KIF20A expression with overall survival (OS) and clinicopathological parameters.

**Results:**

Twenty-five studies involving 7262 patients were finally incorporated, including nine about KIF2A and sixteen about KIF20A. Our results indicated that patients with high expression of KIF2 and KIF20A tended to have shorter OS than those with low expression (HR = 2.23, 95% CI = 1.87–2.65, *P* < .001; HR = 1.77, 95% CI = 1.57–1.99, *P* < .001, respectively). Moreover, high expression of these 2 KIF members was significantly associated with advanced clinical stage (OR = 1.98, 95% CI: 1.57–2.50, *P* < .001; OR = 2.63, 95% CI: 2.03–3.41, *P* < .001, respectively), positive lymph node metastasis (OR = 2.32, 95% CI: 1.65–3.27, *P* < .001; OR = 2.13, 95% CI: 1.59–2.83, *P* < .001, respectively), and distant metastasis (OR = 2.20, 95% CI: 1.21–3.99, *P* = .010; OR = 5.25, 95% CI: 2.82–9.77, *P* < .001, respectively); only high KIF20A expression was related to poor differentiation grade (OR = 1.82, 95% CI: 1.09–3.07, *P* = .023).

**Conclusions:**

High expression of KIF2 and KIF20A in human cancer was significantly correlated with worse prognosis and unfavorable clinicopathological features, suggesting that these 2 KIF members can be used as prognostic biomarkers for different types of tumors. PROSPERO REGISTRATION NUMBER: CRD42019134928.

## 1 Introduction

Cancer is among the most common causes of morbidity and mortality worldwide, particularly in less developed countries.^[1]^ In 2019, nearly 1.76 million new cancer cases are likely to be diagnosed and more than 600,000 patients may die of cancer in the United States.^[2]^ Despite constant efforts to improve the diagnosis and treatment of cancer in recent years, the 5-year survival rate of patients remains unsatisfactory for various types of tumors.^[3]^ This is largely because of inadequate knowledge of the mechanisms that cause cancer and promote disease progression. Thus, studies have focused on evaluating the molecular mechanisms and identifying new biomarkers of cancer to predict prognosis and improve the therapeutic efficacy and survival status of patients with cancer.

The kinesin family (KIF), initially identified by Vale et al in 1985, is present in all eukaryotes and contains 14 super families (more than 40 members).^[4]^ By participating in the polymerization dynamics of microtubules (MTs), KIF can catalyze rapid spatial remodeling of the MT cytoskeleton during the cell cycle, which is necessary for intracellular transport and cell mitosis.^[5,6]^ If any abnormalities occur during cell mitosis, various adverse consequences can occur, such as cell apoptosis, gene mutation, and even cancer development.^[7]^ KIF member 2A (KIF2A), localized to the spindle poles in eukaryotic cells and one of the four members (KIF2A, KIF2B, KIF2C/MCAK, and KIF24) of the kinesin-13 super family, is involved in various biological processes such as cell division, bipolar spindle assembly, and cilia formation.^[6,8]^ Spindles lacking KIF2A cannot separate into bipolar forms in human mitotic cells, which may cause cell cycle arrest or even cell apoptosis.^[9]^ Moreover, KIF2A plays a crucial role in non-mitotic cells. It was reported that KIF2A suppresses the extension of neuronal collateral branches (particularly axons) by depolymerizing MTs.^[10]^ Another KIF member, KIF20A (also known as RAB6KIFL), belonging to kinesin-6 super family, consists of 890 amino acids and is abundantly expressed in the thymus, bone marrow, and testis of adults, with low expression observed in the heart, placenta, and spleen.^[11]^ By interacting with the GTP-bound forms of Rab6, KIF20A can bind to microtubules and generate mechanical force to accelerate the movement of organelles.^[12,13]^ Previous studies showed that KIF20A is essential for cell cycle mitosis and its accumulation promotes the proliferation of both normal and pathological cells.^[14,15]^ Therefore, KIF2A, and KIF20A play important roles in eukaryotic cells.

In recent years, a large number of studies have suggested that abnormal expression of KIF2A and KIF20A is involved in the carcinogenesis of various tumors, such as epithelial ovarian cancer (EOC), breast cancer (BC), colorectal cancer (CRC), and laryngeal squamous cell carcinoma (LSCC).^[16–20]^ These 2 proteins can be used as biomarkers for prognosis of tumors and may play a significant role in cancer-targeted therapy through various molecular mechanisms.^[21]^ Although extensive studies have investigated the correlations between KIF2A and KIF20A expression and different types of cancer, their results have been contradictory because of the small sample size studies and inconsistencies in research methods. Thus, we performed a quantitative meta-analysis to comprehensively assess and accurately analyze the relationship between the expression of these 2 well-known kinesins and survival outcomes and clinicopathological features of patients with cancer.

## 2 Material and methods

### 2.1 Search strategy

According to the PRISMA guidelines, Electronic searches in the PubMed, EMBASE, Cochrane Library, and Web of Science databases were performed by 2 authors independently up to April, 2019. We used the following MeSH terms and related synonym of literature retrieval strategy: (“kinesin family member 2A” OR “KIF2A”) AND (“tumor” OR “cancer” OR “carcinoma” OR “neoplasm” OR “malignancy”) AND (“prognostic” OR “predict” OR “prognosis” OR “survival” OR “outcome”); (“kinesin family member 2A” OR “KIF2A”) AND (“tumor” OR “cancer” OR “carcinoma” OR “neoplasm” OR “malignancy”) AND (“prognostic” OR “predict” OR “prognosis” OR “survival” OR “outcome”). Moreover, manual searches were also conducted through scanning the reference lists of the retrieved articles. Irrelative publications were carefully recruited by scanning the titles, abstracts, keywords, and full texts. Since all analyses were based on previously published studies, ethical approval and informed consent were not needed.

### 2.2 Inclusion and exclusion criteria

Eligible studies should meet the following selection criteria.

Inclusion criteria:

(1) the expression of KIF2A and KIF20A was detected in human cancerous tissues, rather than in any other kinds of specimens;
(2) the association of KIF2A and KIF20A expression with OS was assessed;
(3) sufficiently available survival information was provided for calculating the HR with 95% CIs.

Exclusion criteria:

(1) reviews, letters, comments, meta-analysis, and meeting abstracts;
(2) studies only investigated the molecular mechanism or function of KIF2A and KIF20A;
(3) duplicate publications.

### 2.3 Data extraction

All data were systematically extracted from every study by 2 investigators (Xing Li and Kunpeng Shu) independently, and any disagreements were resolved through discussion with a third reviewer (Zhifeng Wang). Publication information was as follows: name of first author, year of publication, region where the study was performed, cancer type, sample size, adjusted HR and 95% CIs of KIF2A and KIF20A for OS, data extraction method and follow-up time. If both univariate and multivariate analyses were provided by the eligible studies, the latter was directly applied. However, if only Kaplan–Meier curves were available, we extracted data (HR with 95% CI) from the graphical survival plots using the software of Engauge Digitizer 10.8 and the method provided by Tierney et al.^[22]^

### 2.4 Quality assessment

In accordance to the Newcastle-Ottawa quality assessment scale (NOS), study selection, comparability, and outcome were used to assess the quality of recruited studies.^[23]^ The NOS quality scores ranged from 0 to 9, and articles scored greater than 6 were identified as high-quality studies.

### 2.5 Statistical analysis

All the statistical analyses were carried out using the Stata version 14.0 software (Stata Corporation, College Station, TX). We assessed the strength of the relationship between these 2 KIF members with survival outcomes and clinicopathological features through the pooled HR and OR with 95% CIs, respectively. Moreover, a test of heterogeneity among different studies was performed via Higgins *I*^2^ statistics and the chi-square *Q* test, and a random-effects model would be built if the heterogeneity had statistical significance (*P* < .05 or *I*^2^ > 50%). Otherwise, the fixed-effects model was conducted. Probable publication bias was assessed by constructing a funnel plot symmetry and using Begg test. Finally, sensitivity analysis was performed to check the stability of the statistical result. *P* values <.05 were regarded to be statistically significant.

## 3 Results

### 3.1 Study search results

**Figure 1:**
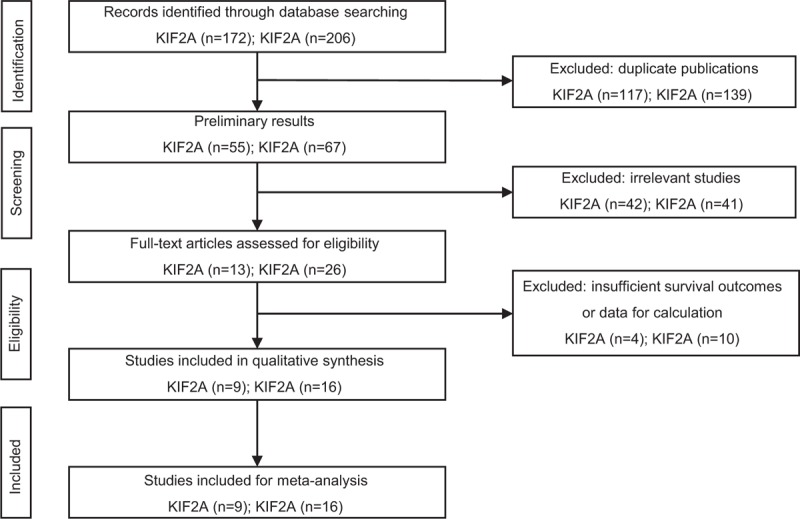
Flow diagram of literature search and selection.

The process flow diagram of literature selection is shown in Figure 1. A total of 378 studies were initially retrieved, including 172 studies of KIF2A and 206 studies of KIF20A. After removing duplicates, 112 papers remained (n = 55; n = 67, respectively). Then, we carefully sifted the titles and abstracts of these articles and excluded 83 irrelevant items (n = 42; n = 41, respectively). Of the remaining articles, 14 were removed owing to lacking of sufficient survival outcomes or data for calculation (n = 4; n = 10, respectively). Finally, 25 studies were included in our meta-analysis (n = 9; n = 16, respectively).

### 3.2 Characteristics of the included studies

The basic characteristics of these studies are summarized in Table 1. In the group of KIF2A, only 1 study was conducted in Japan, while the others were conducted in China, published between 2014 and 2019. Among the included studies, 9 types of tumors were evaluated, including diffuse large B cell lymphoma,^[24]^ lung adenocarcinoma,^[19]^ hepatocellular carcinoma,^[7]^ LSCC,^[25]^ EOC,^[16]^ CRC,^[20]^ lung squamous cell carcinoma,^[26]^ BC,^[27]^ and gastric cancer.^[28]^

**Table 1:**
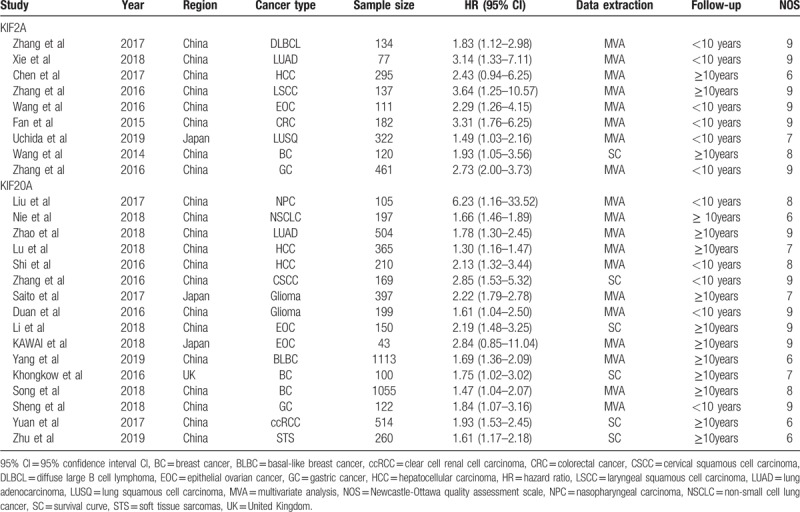
Basic characteristics of the included studies.

In the group of KIF20A, enrolled studies were carried out in 3 countries (12 in China, 2 in Japan, and 1 in the UK), and the publication period ranged from 2016 to 2019. These studies evaluated a total of 11 different types of tumors, including respiratory system carcinoma (1 non-small cell lung cancer and 1 lung adenocarcinoma),^[29,30]^ digestive system carcinoma (2 hepatocellular carcinoma and 1 gastric cancer),^[31–33]^ female reproductive system carcinoma (1 cervical squamous cell carcinoma, 2 EOC and 3 BC),^[17,18,34–37]^ nervous system carcinoma (2 glioma),^[38,39]^ urinary system carcinoma (1 clear cell renal cell carcinoma),^[40]^ and carcinoma of other systems (1 nasopharyngeal carcinoma and 1 soft tissue sarcomas).^[41,42]^

In terms of data extraction, most HRs with 95% CIs were obtained directly from multivariate analysis, and only a quarter of these data points were obtained from survival curves. NOS score of all included studies varied from 6 to 9.

### 3.3 Association between KIF2A and KIF20A expression and OS

**Figure 2:**
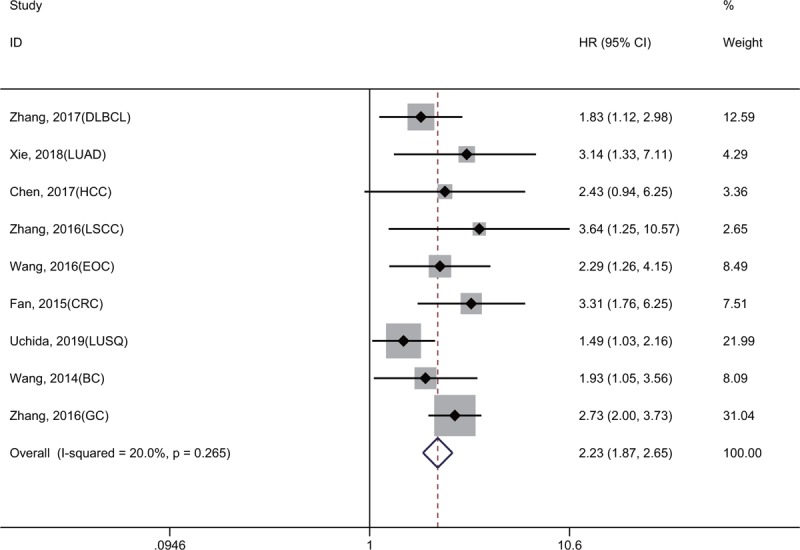
Forest plot reflecting the association between KIF2A and OS.

**Figure 3:**
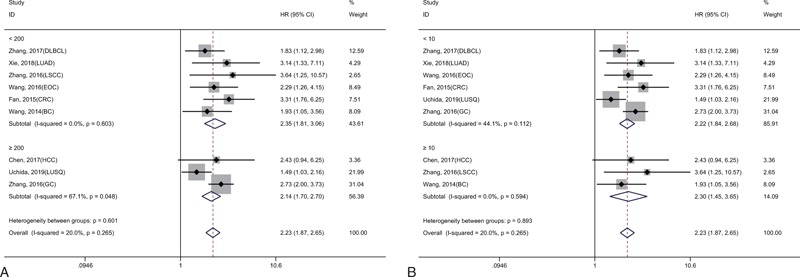
Forest plot showing the subgroup analyses of the pooled HRs with KIF2A in sample size (A) and follow-up time (B).

As shown in Figure 2, the results of 9 studies involving 1839 cancer patients demonstrated that high KIF2A expression was significantly related to a shorter OS (HR = 2.23, 95% CI = 1.87–2.65, *P* < .001). A fixed-effect model was used because no obvious heterogeneity was detected (*I*^2^ = 20.0%, *P* = .265). Moreover, subgroup meta-analyses stratified by sample size (more or less than 200) and follow-up time (more or less than 10 years) were carried out to evaluate the prognostic value of KIF2A (Fig. 3A and B). We found that a sample size greater than 200 or follow-up time of less than 10 years may cause slight heterogeneity, but this did not affect the final conclusion (Table 2).

**Table 2:**
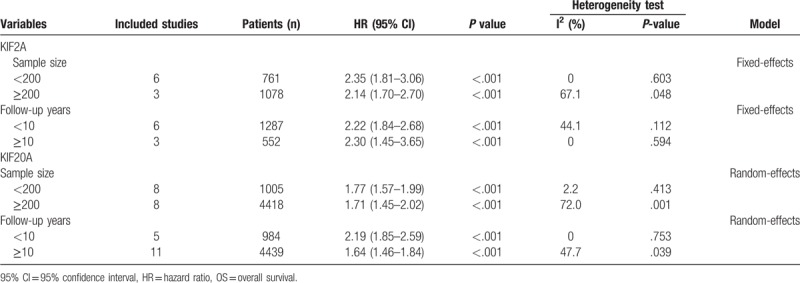
Subgroup analysis of the pooled HR of OS with KIF2A and KIF20A expression in patients with cancer.

**Figure 4:**
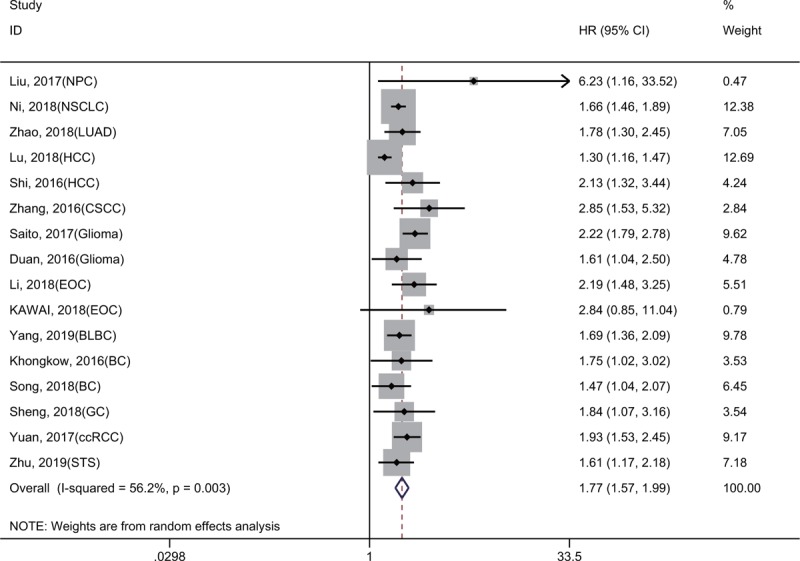
Forest plot reflecting the association between KIF20A and OS.

**Figure 5:**
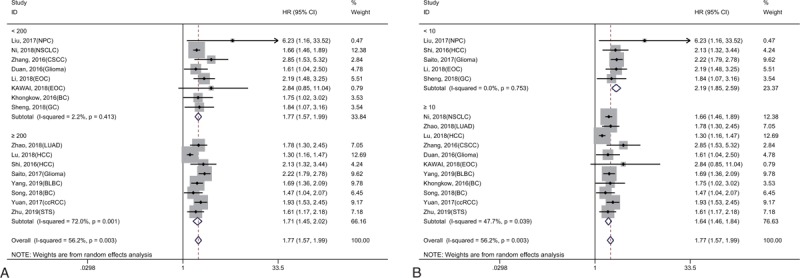
Forest plot showing the subgroup analyses of the pooled HRs with KIF20A in sample size (A) and follow-up time (B).

As shown in Figure 4, 16 studies involving 5423 patients reported an association between KIF20A and OS in different types of tumors. Significant heterogeneity among these studies was found (*I*^2^ = 56.2%, *P* = .003), and thus a random-effect model was used. The results showed that patients with cancer and high KIF20A expression had a significantly poor OS compared to those with low KIF20A expression (HR = 1.77, 95% CI = 1.57–1.99, *P* < .001). We then conducted subgroup meta-analyses to assess whether the heterogeneity was related to the sample size and follow-up time (Fig. 5A and B). The results revealed that a sample size greater than 200 or follow-up time of greater than 10 years may be the main source of heterogeneity, but this did not affect the conclusion (Table 2).

Overall, high expression of KIF2A and KIF20A was associated with shorter OS, and these two KIF members can serve as independent factors for predicting the survival outcomes of patients with cancer.

### 3.4 Association between KIF2A and KIF20A expression and clinicopathological features

**Figure 6:**
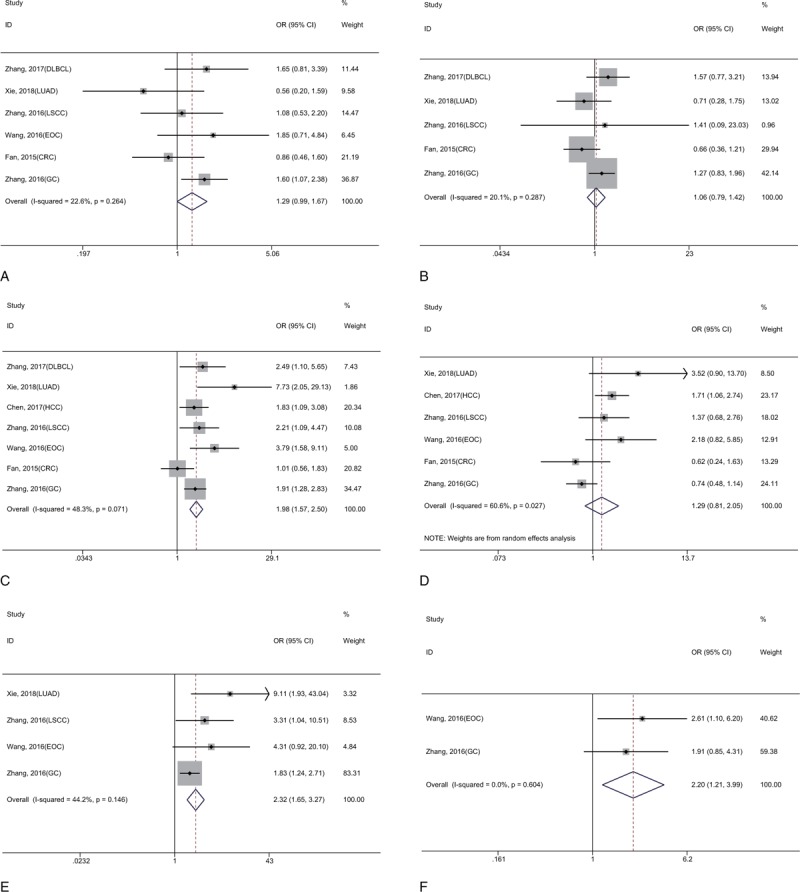
Forest plot reflecting the association between KIF2A and clinicopathological features (A, Age; B, Gender; C, Clinical stage; D, Differentiation grade; E, LNM; F, DM).

**Figure 7:**
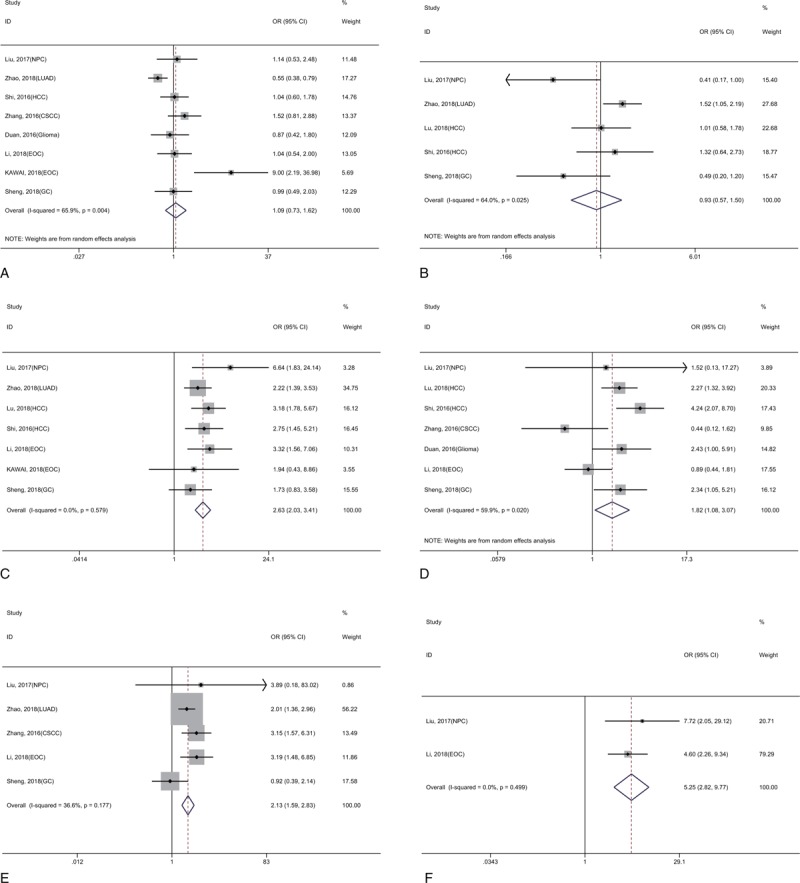
Forest plot reflecting the association between KIF20A and clinicopathological features (A, Age; B, Gender; C, Clinical stage; D, Differentiation grade; E, LNM; F, DM).

Not all enrolled studies fully recorded the correlation of KIF2A and KIF20A expression with clinicopathological features, and the main clinicopathological features in our meta-analysis included age, gender, clinical stage, differentiation grade, lymph node metastasis (LNM), and distant metastasis (DM) (Table 3). The fixed-effects model or random-effects model was built according to whether significant heterogeneity was present. As presented in Figures 6 and 7, there was no remarkable correlation between KIF2A and KIF20A expression and age (OR = 1.29, 95% CI: 0.99–1.67, *P* = .053; OR = 1.09, 95% CI: 0.73–1.62, *P* = .674, respectively), gender (OR = 1.06, 95% CI: 0.79–1.42, *P* = .710; OR = 0.93, 95% CI: 0.57–1.50, *P* = .752, respectively). However, high expression of these 2 proteins was significantly associated with an advanced clinical stage (OR = 1.98, 95% CI: 1.57–2.50, *P* < .001; OR = 2.63, 95% CI: 2.03–3.41, *P* < .001, respectively), positive LNM (OR = 2.32, 95% CI: 1.65–3.27, *P* < .001; OR = 2.13, 95% CI: 1.59–2.83, *P* < .001, respectively), and presence of DM (OR = 2.20, 95% CI: 1.21–3.99, *P* = .010; OR = 5.25, 95% CI: 2.82–9.77, *P* < .001, respectively), and only high KIF20A expression was related to poor differentiation grade (OR = 1.82, 95% CI: 1.09–3.07, *P* = .023).

**Table 3:**
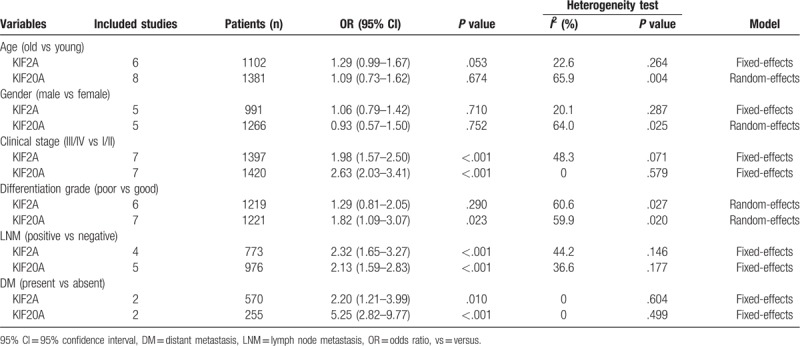
Association between KIF2A and KIF20A expression and clinicopathologic features.

### 3.5 Publication bias and sensitivity analysis

**Figure 8:**
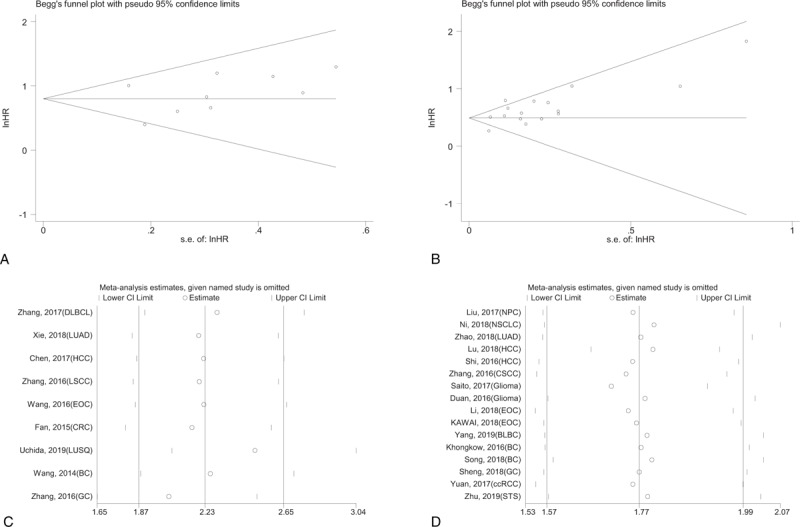
Detection of publication bias for meta-analysis of OS (A, KIF2A; B, KIF20A), and sensitivity analysis of the relationship between KIF2A and KIF20A expression and OS (C, KIF2A; D, KIF20A).

As shown in Figure 8A and B, Begg was used to assess the publication bias of the studies included in our meta-analysis. No significant bias was observed in analysis of the association between KIF2A and KIF20A expression and OS (P_KIF2A_ = .348, P_KIF20A_ = .163). Sensitivity analysis was conducted by excluding each single study, and no single study affected the final conclusions (Fig. 8C and D). Therefore, the summarized results of our meta-analysis were relatively stable and reliable.

## 4 Discussion

Cancer remains a major public health problem worldwide, and the overall incidence and mortality rates have increased in recent years.^[2]^ Although numerous studies have examined cancer pathogenesis, most patients are diagnosed at an advanced stage and show poor survival outcomes because of a lack of early diagnostic and prognostic biomarkers.^[43]^ Recently, KIF has gained attention in cancer research because of its role in the transport of mRNAs, protein complexes, and organelles during cell mitosis and meiosis.^[6]^ Mutations in some members of KIF may lead to congenital and hereditary diseases, including cancer.^[44]^ An increasing number of studies has shown that members of KIF, particularly KIF2 and KIF20A, are involved in cell differentiation, proliferation, invasion, and migration during the course of cancer, and can be used as biomarkers for cancer prognosis. Therefore, we performed this meta-analysis of eligible studies to further examine the prognostic significance of KIF2A and KIF20A in different types of human cancers.

We identified 25 recently published articles and found that patients with cancer and high KIF2 and KIF20A expression tended to have shorter OS than those with low expression. Thus, these 2 KIF members can be used as biomarkers for cancer prognosis. Moreover, subgroup analysis was conducted to explore the correlation between HRs and different variables, including sample size and follow-up time, which are the main sources of heterogeneity. The results also indicated that high expression of these 2 proteins was significantly related to unfavorable clinicopathological features, such as advanced clinical stage, poor differentiation grade, positive LNM, and presence of DM. No previous meta-analysis has comprehensively evaluated the relationship of KIF2A and KIF20A expression with the prognosis and clinicopathological features in different types of tumors. This is the first meta-analysis to demonstrate the prognostic value of these 2 proteins in human cancer.

In 2010, Wang et al first reported that KIF2A, as an MT depolymerase, was expressed at higher levels in squamous cell carcinoma of the oral tongue cells than in paracancerous tissues, and its overexpression predicted a high lymph nodal metastatic rate and advanced tumor clinical stage.^[45]^ Four years later, a study of 120 female patients confirmed the prognostic value of KIF2A in BC, demonstrating that patients with higher KIF2A expression have worse survival outcomes.^[27]^ However, as early as in 2005, Taniuchi and colleagues demonstrated that KIF20A (RAB6KIFL) is involved in pancreatic ductal adenocarcinoma and can serve as a candidate target for drug discovery for treating this cancer type at the molecular level.^[46]^ Subsequently, Lu et al found that the expression of some genes, including KIF20A, was commonly up-regulated in bladder tumors in both humans and rodents.^[47]^ Furthermore, in a study of 169 patients with cervical squamous cell carcinoma, Zhang et al revealed that KIF20A expression was significantly related to aggressive clinicopathological features and is an independent biomarker for predicting the survival outcomes of patients with this cancer.^[34]^ After that, the prognostic value of these two proteins has been continuously confirmed in various cancers.

The expression mechanisms of KIF2A and KIF20A are different and complicated in various types of cancer. First, these 2 proteins can induce cancer cell proliferation, apoptosis and migration through regulating various signaling pathways, such as the phosphatidylinositol-3-kinase (PI3K)/protein kinase B (Akt) signaling pathways, and the E2F-retinoblastoma protein-p16 pathway.^[15,48]^ Second, the expression of these 2 proteins can be suppressed by certain miRNAs, which may provide candidate novel molecular targets for precise cancer treatment.^[26,49]^ Finally, some cancer-related proteins or enzymes are involved in regulating the expression of KIF2A and KIF20A. For instance, down-regulation of KIF2A in gastric cancer inhibits tumor cell invasion through suppressing the expression of Membrane type 1-matrix metalloproteinase.^[50]^ Besides, forkhead box protein M1 can enhance the radiation resistance of lung cancer through inducing KIF20A expression.^[51]^ Several studies have been carried out to explore the biological functions of KIF2A and KIF20A in various tumor cells, but the molecular mechanisms underlying these 2 proteins and cancer progression remain unclear so far.

Notably, KIF2A and KIF20A not only have potential as useful biomarkers for predicting prognosis, but also can serve as novel and potential therapeutic targets in various malignant tumors through different mechanisms.^[21]^ In 2013, a Japanese scientific research team performed a single-center phase I/II clinical trial of immunotherapy and developed a pancreatic cancer vaccine, KIF20A-66, composed of human leukocyte antigen human leukocyte antigen-A24-restricted epitope peptide derived from KIF20A.^[52]^ This vaccine significantly prolonged the OS of patients. The reliable effects of the KIF20A-derived peptide were confirmed in various advanced digestive system cancers through clinical trials.^[53–55]^ Khongkow et al also found that if KIF20A expression was inhibited in vivo, the sensitivity of BC to alkylator-based chemotherapy was increased.^[36]^ Similarly, Wang and colleagues reported that gene silencing of KIF2A suppressed cell proliferation and improved the anti-tumor effect of 5-fluorouracil in squamous cell carcinoma of the oral tongue, suggesting that KIF2A can be developed as a drug target for treating this cancer type.^[56]^ Therefore, further studies of the carcinogenesis mechanism of KIF2A and KIF20A may lead to the development of molecule-targeted drugs and benefit patients with cancer.

## 5 Limitations

There were some limitations to this study. First, we may have omitted some relevant papers because of the limited number of databases searched. Second, most studies included in our meta-analysis were carried out in Asia, which may affect the applicability of the results in western countries. Third, the cut-off values, which were used to distinguish between high and low groups of KIF2A/KIF20A expression, were difficult to define by the uniform criteria because of the diversity of different cancer types, which may have led to some heterogeneity in the results. Finally, the sample sizes of eligible studies were relatively small, with only 9 studies of KIF2A including 1839 patients and 16 studies of KIF20A including 5423 patients finally included in the present meta-analysis. Thus, further large-scale, high-quality, and better designed multi-center studies should be performed to clarify the function of KIF2A and KIF20A in various human cancers.

## 6 Conclusion

This meta-analysis revealed that high expression of KIF2A and KIF20A was significantly correlated with poor OS and adverse clinicopathological features of patients with different types of cancer, indicating that these 2 KIF members can serve as unfavorable prognostic factors and novel therapeutic targets for human cancers. However, further comprehensive studies should be conducted to confirm these results and provide accurate guidance for clinicians in prognosis assessment and individualized treatment of patients with cancer.

## Acknowledgments

We gratefully acknowledge the time and energy contributed by all researchers.

## Author contributions

**Conceptualization:** Xing Li.

**Data curation:** Xing Li, Kunpeng Shu, Zhifeng Wang.

**Formal analysis:** Xing Li, Kunpeng Shu, Zhifeng Wang.

**Funding acquisition:** Degang Ding.

**Investigation:** Xing Li, Kunpeng Shu, Zhifeng Wang, Degang Ding.

**Methodology:** Xing Li, Kunpeng Shu, Zhifeng Wang, Degang Ding.

**Project administration:** Degang Ding.

**Resources:** Degang Ding.

**Software:** Xing Li, Kunpeng Shu.

**Supervision:** Zhifeng Wang, Degang Ding.

**Validation:** Xing Li, Kunpeng Shu, Zhifeng Wang, Degang Ding.

**Visualization:** Xing Li, Kunpeng Shu, Zhifeng Wang, Degang Ding.

**Writing – original draft:** Xing Li, Kunpeng Shu, Zhifeng Wang, Degang Ding.

**Writing – review & editing:** Xing Li, Kunpeng Shu, Degang Ding.
